# Where exactly do the social and behavioural sciences fit in One Health?

**DOI:** 10.3389/fpubh.2024.1386298

**Published:** 2024-05-15

**Authors:** Lucy Carter, Aditi Mankad, Walter Okello

**Affiliations:** ^1^CSIRO Environment, Brisbane, QLD, Australia; ^2^CSIRO Environment, Canberra, ACT, Australia

**Keywords:** behavioural sciences, one health, disciplinary integration, social sciences, humanities, cooperation

## Abstract

At its core, One Health promotes multidisciplinary cooperation amongst researchers and practitioners to improve the effectiveness and management of complex problems raised by the interplay of human, animal and environment interactions. Contemporary One Health literature has identified reducing disciplinary barriers as key to progress in the field, along with addressing the notable absence of social sciences from One Health frameworks, among other priorities. Efforts to position social scientists as experts on behaviour change and health decision-making has helped to articulate a concrete role for progressing One Health collaborations. Yet, there are other equally valuable functions the social scientist has in understanding complex systems, like One Health. We make explicit the multiple and diverse knowledge contributions the social sciences and humanities can make to progressing the One Health agenda. Articulating these more clearly invites a broader set of interdisciplinary perspectives to One Health discussions, allowing for stronger connections between sectors, actors, disciplines, and sub-systems. This perspective piece identifies a range of entry points for researchers and practitioners to better utilize the potential contributions social sciences and humanities scholars can make to One Health goals.

## Situating the social and behavioural sciences as key partners in One Health

There exists both narrow and broad definitions of One Health (OH) which draw upon a mix of global movements, disciplinary integration efforts and socio-ecological concepts to improve environmental, animal and human health (1–5). As a movement, OH is relatively new (*circa* 2000s), largely emerging as a response to the rise of global pandemics such as the SARS virus, and Ebola (6). However, the foundations of OH philosophy (i.e., the apparent links between animal and human medicine and the existence of social and political determinants of disease) can be traced back to 19th century pathologist Rudolf Virchow, and Calvin Schwabe’s ‘One Medicine’ concept of the 1980s (7). Consistently championed by the veterinary profession, adoption of OH approaches now appear in the international strategies of the Food and Agriculture Organization and the World Health Organization (6).

While historically OH has enjoyed firm footing in biosecurity and public health contexts, OH frameworks continue to emerge more broadly across other complex socio-ecological settings including sustainable livelihoods (8, 9), enhancement of food and nutrition security (10), and non-communicable disease (6). This shift has enabled a broader set of social and interdisciplinary science experts to become involved in applying OH concepts to better articulate complexity, despite these broader applications of OH occurring at a distance from the mainstream.

While reference to the social sciences in OH is not new, there are renewed calls for its stronger and more explicit inclusion in OH frameworks and programming (3, 11). The ‘social sciences’ label has not always been formalized in OH agendas, nor consistently defined (15). In current OH literature, debate on how and when to utilize the social sciences in how OH is conceptualized, coordinated, implemented and evaluated, has deepened (12).

In principle, *social science* is a branch of science that examines human and societal behaviours, interactions, and structures. Commonly treated as a single discipline, the social sciences are a collection of distinct disciplinary streams, each housing a multitude of subdisciplines. From about the 1950s, the term *behavioural science* began to emerge to describe disciplines with more quantitative and experimental methodologies (e.g., psychology and economics), distinguishing these from other social science disciplines that relied on more qualitative or observational methods (13).

Despite the breadth of social and behavioural sciences available to OH, to date, only select characteristics of a small number of disciplines readily appear in OH discussions and these typically fall into the behavioural science family (11, 14). In this paper we contend that consideration of a much broader spectrum of social and behavioural sciences capability will better assist OH to meet its contemporary goals.

While the overarching goal of OH is to achieve optimal health for people, animals, plants, and the environment, multiple knowledge and practice gaps have been identified before progress toward this goal can be realized. Among these gaps, sometimes referred to as priorities, are included: improved conceptualization and programming to address system complexity; strengthened and more diverse empirical data collection and use; and enhanced sectoral collaboration and policy coordination (1, 3, 12, 15).

Additionally, the normative aspects of knowledge production and use in OH including efforts to decolonize research approaches and recognize the legitimacy of local and Indigenous knowledge systems has been identified as an outstanding critical theme to address (3, 14, 16).

In combination, the social and behavioural sciences are perfectly placed to assist OH in addressing these priorities. Yet, OH planners and implementers will first need to reconsider how they continue to perceive and utilize this capability in general. Social scientists share some responsibility for addressing this challenge and can themselves assist with advocating for their broader inclusion by providing both clarity and direction. This paper goes someway to addressing the challenge at hand.

Below, we make explicit the multiple and diverse knowledge contributions the social and behavioural sciences can make to progressing the OH agenda. In addition, we advocate for the inclusion of humanities scholarship to be recognized as a potentially valuable partner in the quest to advance OH outcomes. For the remainder of this paper, we use the overarching term *social and behavioural sciences* (SBS) to describe the broad area of science that we consider critical to advancing the OH agenda.

## Conventional social and behavioural science contributions to One Health

The motivation to reconsider the full contributory value of the SBS is largely attributable to the realization that both global and local drivers for infectious disease emergence and resolution are complex and interrelated (1). Dynamic global change like geopolitical instability, increased urbanization, the breakdown of health systems, and impacts of climate change are now recognized as likely influences on human-animal-environment health dynamics (1, 17, 18). A lack of significant progress in meeting OH goals may be another driver to revisit the value of broader multidisciplinary cooperation (19).

*Prediction* of human response to risk, for example, is arguably the most recognized contribution of the SBS to OH. Presently, a highly visible (and valued) SBS contribution to OH involves psychological science, especially as it relates to risk management, risk communication and behaviour change. Often transpiring as knowledge, attitude and practice (KAP) studies, use of these methods has a reputation for producing quantitative information quickly, a characteristic especially useful in fast-moving outbreak contexts (11, 20).

Equally, economics has widely been used to quantify the cost of OH diseases and less frequently to model the effectiveness of OH interventions, often due to methodological challenges. Ascertaining the efficiency of resource allocation is a key undertaking in economics and could be a major contribution of economics in OH planning, implementation, and evaluation if the technical challenges can be overcome (21–23). Arguably, these applications of behavioural science can deliver useful information for OH agendas, but they can overlook critical information that might for example, generate insights about context, conflict, and complex institutional relationships.

Aside from prediction and quantification, the social sciences can be applied to *interpret*, to *empower*, and to *deconstruct*, depending on one’s philosophical position on how knowledge is acquired and treated (24). In this sense, anthropology has historically enjoyed some engagement with OH, although its recognition as a key partner in OH is also yet to be fully realized (25).

There are various other facets of OH where stronger collaboration with a broader range of disciplinary knowledge sets would benefit. Understanding embedded cultural and institutional norms in disease contexts; or the effects of institutional dynamics on health governance; or the macro drivers of broader systems interactions, are all potentially valuable knowledge dimensions to OH advancement (16). For these data points, deeper engagement with sociology, anthropology, human geography, sustainability science, and other social science disciplines, which more readily employ qualitative methodologies for example, may be more conducive to answering a broader range of research questions.

## Engaging the humanities as a valuable partner

An additional and important group of disciplines whose value is also yet to be fully realized in OH is the humanities (21). Not easily fitting under the social sciences label, ethicists, historians, philosophers, educators and legal scholars have distinct capabilities to enhance our understanding of health policy, health governance, and the broader institutional influences on health outcomes (26, 27). Often possessing skills in non-empirical methods, this group of experts can be instrumental in sharpening the theoretical, critical, and analytical aspects of OH research and practice.

## The broad and varied disciplinary contributions possible

Moving beyond common representations of social scientists as mostly comprising of psychologists, science communicators, and economists, and adding humanities scholarship to the pool of valuable expertise, potentially unlocks the considerable capability needed to meet contemporary OH priorities. Table 1 compiles examples of attributes from a broader set of SBS and humanities scholars. These attributes are aligned to seven key goals, sometimes identified as knowledge gaps, or priorities of focus needed to advance the OH agenda.

**Table 1:** Aligning the varied skillsets, methods and techniques held by social and behavioural scientists and humanities scholars, to address current One Health priorities.

Contemporary One Health priorities	Social and behavioural sciences and humanities capabilities to strengthen One Health outcomes (as examples)
Advance empirical knowledge of human, social and institutional dimensions	Apply research methods from diverse epistemological origins to enhance understanding of human-animal-environment system interactions as well as impacts of OH diseases and conditions on these systems; Identify actors’ diverse goals, priorities, motivations, and perceptions of and exposure to risk; Identify how formal and informal institutions (norms, cultures, practices, policies, regulations, markets) and social relationships frame responses to risk and action (1, 3, 24, 28).
Secure the integrity of knowledge production and use	Reveal researcher positionality and its influence on research framing; Highlight the structural inequalities and power dynamics that influence agency, decision-making and behaviour; Assist to decolonize knowledge production and use, bring local and Indigenous knowledge to the fore; Guide the navigation of ethical research processes (14, 15, 19, 21, 29).
Reduce disciplinary and sectoral divisions for improved outcomes	Promote, support and facilitate cross-disciplinary and transdisciplinary processes to bring together a broader spectrum of actors; Apply knowledge and partnership brokering processes to dynamic contexts; Inform multistakeholder coordination and policy alignment to strengthen sectoral collaboration; Engage with diverse actors and stakeholders to facilitate change processes (11, 20, 21).
Refine the One Health agenda; inform goal setting	Elevate social and behavioural scientific and human dimensions to better inform OH frameworks and agendas; Promote cross-learning with other complimentary approaches (e.g., systems approaches); Reframe OH goals to include systems dynamics, actor relationships, and institutional drivers more explicitly (14, 16).
Design and facilitate learning processes	Apply monitoring, evaluation and learning (MEL) frameworks to the design and implementation of OH interventions; Identify the ethical engagement and practice considerations of research framing, of intervention design, and evaluation strategies (11, 16).
Differentiate scale, sector and system for greater clarity and impact	Reveal human and social interactions and relationships across scales and systems for improved OH outcomes; Expand the focus of OH intervention design to include policy level considerations and intergovernmental collaboration (30).
Expand the methodological toolbox	Bring “deep” qualitative methodologies and approaches to the current methods mix; Highlight outcomes and impacts of system change, and distribution of costs, benefits, risks [cf. (31)].

The priorities span the research and practice spectrum and include: advancing empirical knowledge; ensuring integrity of knowledge production; reducing barriers to collaboration; refining the OH agenda and its framing; designing and facilitating learning processes; differentiating scale, sector and system; and expanding the methodological toolbox (1, 3, 12, 15).

In developing Table 1, we have resisted the urge to label individual disciplines as having the responsibility (or the expertise) for the skillset combinations identified here for two reasons. First, in complex problem settings, we recognize *sub*-disciplines can play a leading role in knowledge production and practice, especially in specific disease contexts. Second, we believe that branding one discipline as the gatekeeper or holder of a specific skillset would be misleading given our own knowledge limitations of the full breadth and depth of the social and behavioural sciences family.

We also acknowledge there is likely overlap across a number of these capabilities and skillsets and their interpretation and translation might differ according to the disciplinary lens applied. Table 1 presents examples of potential capabilities on offer through broader and deeper engagement with SBS and the humanities.

## The significance of better integrated OH approaches

Two topical examples of the value of engaging more fully with the SBS and humanities when attempting to apply a more integrated OH approach include the global COVID-19 pandemic and the 2021 New South Wales mouse plague. In the case of COVID-19, ongoing societal impacts of health system pressures, the prevalence of social and mental health consequences of ‘long covid’, and continued disruption to global trade and transport systems provide clear entry points for cross-disciplinary collaboration beyond typical OH expertise (32). The COVID-19 pandemic demonstrated the necessity of cooperation to enable multisectoral responses across academia, civil society, government, and private enterprise including at multiple levels (33). The task of coordinating information across disciplinary silos was compounded by the urgency with which responses were required. Adding to this challenge were considerable gaps in global knowledge about disease transmission pathways. This complexity demonstrates the need for better integrated OH approaches incorporating the full contribution SBS and humanities scholars can make to health outcomes.

In the case of Australian mouse plagues, the full extent of the socio-economic and community impacts of mouse outbreaks on regional and rural communities is visible, but yet to be formally described (34, 35). Research is ongoing to measure the type, extent and severity of impacts of recent mouse outbreaks on Australian individuals and communities. Yet, international evidence suggests the prevalence of significant mental stress, often exacerbated by social disadvantage, has contributed to poor mental and social health consequences during rodent invasion (35). In addition, Australian media reports continue to draw parallels between the lived experience of mouse plagues and the aftermath of natural disasters [cf. (36, 37)]. As in the case of COVID-19, much broader systemic interactions which extend beyond zoonotic spread, and into social, cultural, political, and economic spheres, are yet to emerge [cf. (34)].

## Where next?

In the same way that biophysical sciences can model health and disease spread to respond to global health threats, the social sciences in OH have largely been applied to predict human behavioural responses to events and to have influence on both prevention and management. There are however many more potentially valuable contributions that SBS and humanities scholars are able to offer OH. We have provided examples of some of these capabilities and attributes here, many of them suited to addressing issues relating to systems complexity, responsible science practice, and multisectoral cooperation – all identified as important to OH progress.

There are likely to be other possible SBS contributions valuable to OH that we have not identified here. While the OH community strives to better engage the broader SBS community to explore points of intersection, it is also the task of SBS and humanities scholars to assist the OH community to identify these connections. All camps will need to be present if cross-disciplinary integration is to achieve the impact it seeks in advancing the OH agenda.

## Data availability statement

The original contributions presented in the study are included in the article/supplementary material, further inquiries can be directed to the corresponding author.

## Author contributions

LC: Conceptualization, Investigation, Writing – original draft, Writing – review & editing. AM: Conceptualization, Writing – original draft, Writing – review & editing. WO: Conceptualization, Writing – original draft, Writing – review & editing.
